# Extensive Contact Lens Wear Modulates Expression of miRNA-320 and miRNA-423-5p in the Human Corneal Epithelium: Possible Biomarkers of Corneal Health and Environmental Impact

**DOI:** 10.3390/genes15060816

**Published:** 2024-06-20

**Authors:** Anna M. Roszkowska, M’hammed Aguennouz, Emanuela Aragona, Romana Gargano, Giovanni William Oliverio, Leandro Inferrera, Pasquale Aragona

**Affiliations:** 1 Ophthalmology Unit, Department of Biomedical Sciences, University Hospital of Messina, 98125 Messina, Italy; giovanniwilliam.oliverio@unime.it (G.W.O.); inferreraleandro@gmail.com (L.I.); paragona@unime.it (P.A.); 2 Ophthalmology Department, Faculty of Medicine and Health Sciences, Andrzej Frycz Modrzewski Krakow University, 30-705 Kraków, Poland; 3 Unit of Neurology and Neuromuscular Diseases, Department of Clinical and Experimental Medicine, University of Messina, 98122 Messina, Italy; aguenoz.mhommed@unime.it; 4 IRCCS San Raffaele Scientific Institute, Ophthalmology Clinic, Vita Salute San Raffaele University, 20132 Milan, Italy; aragona.emanuela@hsr.it; 5 Department of Economics, University of Messina, 98122 Messina, Italy; romana.gargano@unime.it; 6 Eye Clinic, Department of Medical, Surgical Sciences and Health, University of Trieste, 34127 Trieste, Italy

**Keywords:** miRNA, contact lens wear, corneal epithelium, biomarkers, dry eye

## Abstract

The identification of new biomarkers of ocular diseases is nowadays of outmost importance both for early diagnosis and treatment. Epigenetics is a rapidly growing emerging area of research and its involvement in the pathophysiology of ocular disease and regulatory mechanisms is of undisputable importance for diagnostic purposes. Environmental changes may impact the ocular surface, and the knowledge of induced epigenetic changes might help to elucidate the mechanisms of ocular surface disorders. In this pilot study, we investigated the impact of extensive contact lens (CL) wearing on human corneal epithelium epigenetics. We performed ex vivo analysis of the expression of the miR-320 and miR-423-5p involved in the processes of cellular apoptosis and chronic inflammation. The human corneal epithelium was harvested from healthy patients before the photorefractive keratectomy (PRK). The patients were divided into two age- and sex-matched groups accordingly to CL wearing history with no CL wearers used as a control. The epithelium was stored frozen in dry ice at −80 °C and forwarded for miRNA extraction; afterwards, miRNA levels were detected using real-time PCR. Both miRNAs were highly expressed in CL wearers (*p* < 0.001), suggesting epigenetic modifications occurring in chronic ocular surface stress. These preliminary results show the relationships between selected miRNA expression and the chronic ocular surface stress associated with extensive CL use. MicroRNAs might be considered as biomarkers for the diagnosis of ocular surface conditions and the impact of environmental factors on ocular surface epigenetic. Furthermore, they might be considered as new therapeutic targets in ocular surface diseases.

## 1. Introduction

MicroRNAs (miRNAs) are defined as non-coding, small RNAs of approximately 22 nucleotides that act as post-transcriptional regulators. It is accepted that a human being has about 1900 miRNA sequences, which control from 30 to 50% of gene expression, mostly functioning as gene suppressors, and are involved in several physiological and pathological metabolic pathways [1,2,3].

Since miRNAs are very resistant to degrading enzymes such as RNAses, and since they were found in all cells and in some body fluids (e.g., blood, urine, saliva, tears), they might be studied as diagnostic biomarkers [2,3].

In recent years, microRNAs have attracted a lot of attention in the fields of tumors [4,5], retinal pathologies [6], ocular surface inflammatory processes and overall dry eye diseases [7,8].

The cornea, together with the other structures of the ocular surface, is responsible for protection from environmental agents [3]. Especially, the corneal epithelial cells play a significant role in repairing processes and their changes are described in several ocular surface diseases [3,7,8,9,10].

Recent studies demonstrated the involvement of miRNAs in the pathogenesis of ocular surface diseases, such as dry eye disease (DED) and Sjögren Syndrome (SS), and miR-328 could protect corneal cells and promote re-epithelialization in the DED treatment [6].

The study of miRNAs in ocular surface disorders can contribute to understanding the mechanisms of diseases, and potentially guide the diagnosis and treatment [3,7,8,9,10,11].

Furthermore, corneal epithelial cell changes have been correlated to external environment exposure, such as cigarette smoke, particulate matter, and contact lens use [12,13,14,15,16]. Indeed, contact lens-induced trauma to the corneal epithelium results in an increase in intracellular reactive oxygen species (ROS) production and loss of mitochondrial transmembrane potential. The induced oxidative stress causes the dysfunction of mitochondria, which may trigger mitochondrial pathways of apoptosis in corneal epithelial cells and keratocytes [17,18,19].

Additionally, oxidative stress induces ubiquitin proteasome system impairment, activating selective autophagy for the degradation of misfolded and damaged proteins.

Autophagy is a cytoprotective phenomenon that facilitates cell survival under stressful conditions such as hypoxia and metabolic stress, maintaining the homeostasis and transparency of the cornea [17,18,19]. Much evidence has demonstrated that miRNAs have a double function in autophagy regulation, including anti-autophagy and pro-autophagy roles [19].

MiR-320 was found to regulate the metabolic pathway of the apoptotic process targeting TRIAP1, which is involved in the control of the mitochondrial apoptotic pathway by ensuring the accumulation of cardiolipin in mitochondrial membranes and interacting with several proteins and complexes [20].

MiR-423-5p was found to control critical pathways involved in inflammatory processes, regulating NFAM1 and the protein NFAT, a type I membrane receptor that activates cytokine such as the IL-13 and TNF-α, also regulating the signaling and development of B-cells [21].

Previous studies demonstrated the upregulation of miR-320 and miR-423-5p in various ocular diseases such as diabetic retinopathy, in which superoxide radicals are involved in the uncoupling of mitochondrial electron transport chains, resulting in oxidative stress, inflammation and cell apoptosis [21].

Considering these miRNA characteristics, the aim of this study is to investigate the expression of miR-320 and miR-423-5p in the corneal epithelium cells of healthy contact lens wearers to determine their possible role as potential biomarkers of corneal epithelial and ocular surface changes induced by chronic stress.

## 2. Materials and Methods

The prospective experimental study involved 39 healthy patients (15 M, 24 F) who underwent laser refractive surgery with photorefractive keratectomy (PRK) at the Refractive Surgery Unit of the Ophthalmology Clinic of the University of Messina, Italy. The patients with history of systemic diseases, corneal or ocular surface diseases, allergic conjunctivitis, dry eye disease and systemic and/or local therapies, and previous ocular surgery were excluded from the study. Corneal topography (Antares^®^, CSO, Scandicci, Florence, Italy) and tomography (Pentacam^®^, Oculus, Wetzlar, Germany) were performed in all subjects before the surgery to exclude the corneal diseases that constitute contraindications for the laser procedure. The topographer was also used for Non-Invasive Break Up Time (NIBUT) assessment to exclude the subjects with ocular surface alterations. Only patients with preoperative NIBUT superior to 10 s were enrolled.

The patients were divided into two groups in relation to the contact lens (CL) wearing. Group 1 included subjects with a history of the extensive use of disposable daily, weekly or monthly CL for at least 8 h a day during the last 5 years. All types of disposable CL were included in this study as their effect on the ocular surface was found to produce similar effects [22]. The CL was suspended for 7–10 days prior to the surgery. Group 2 comprised individuals who did not use CL and was used as a control. The corneal epithelium was removed before the PRK was used for analysis.

This study was approved by the Ethical Committee of University Hospital of Messina and was conducted in accordance with the tenets of the Declaration of Helsinki. The informed written consent to collect epithelium for scientific purposes was obtained from all subjects after an explanation of the nature of this study.

### 2.1. Corneal Epithelium Collection

Prior to the standard excimer laser PRK, the oxibupivacaine drops (Alfa Intes, Casoria, Italy) were administered 4 times each 10 min and the corneal epithelium was harvested mechanically with a blunt spatula from the corneal area with diameter of 9 mm. The collected epithelium was immediately stored frozen dry ice at −80 °C and forwarded to the laboratory for the miRNA extraction.

The corneal epithelium from only one eye of each subject was used for miRNA investigation.

### 2.2. Real-Time PCR for miRNAs

#### 2.2.1. microRNA Extraction

Enriched microRNAs were extracted from frozen stored corneal epithelium in dry ice at –80 °C, using mirVana™ miRNA Isolation kit (Ambion, Thermo Fisher, Milan, Italy), and Total RNA was extracted too using a Total Nucleic Acid Isolation kit (Ambion) following the manufacturer’s protocol. The concentrations of samples were measured spectrophotometrically using a Bioanalyzer tool (Agilent Technologies, Santa Clara, CA, USA).

#### 2.2.2. Reverse Transcriptase Reactions

Reverse transcriptase reaction contained RNA samples including purified miRNA, 50 nM stem-loop RT primer of each miRNA (RNU6, miR320 and miR-423-5p) purchased from Thermo Fisher, Milan, Italy, 0.25 mM each of dNTPs, 3.33 U/μL MultiScribe reverse transcriptase (P/N: 4319983, Life Technologies, Carlsbad, CA, USA) and 0.25 U/μL RNase inhibitor (P/N: N8080119; Life Technologies). The 7.5 μL reactions were incubated in a thermocycler for 30 min at 160 °C, 30 min at 420 °C and 5 min at 850 °C and then held at 40 °C. All reverse transcriptase reactions, including no-template controls and RT minus controls, were run in duplicate.

#### 2.2.3. Real-Time PCR

Real-time PCR was performed using a standard TaqMan PCR kit protocol (Thermo Fisher, Milan, Italy) on an Applied Biosystems (Waltham, MA, USA) 7300. The 10 μL PCR included 0.67 μL RT product, TaqMan Universal PCR Master Mix (P/N: 4324018, Life Technologies), 0.2 μM TaqMan probe. The reactions were incubated in a 96-well plate at 95 °C for 10 min, followed by 40 cycles of 95 °C for 15 s and 60 °C for 1 min. All reactions were run in triplicate. The threshold cycle (CT) is defined as the fractional cycle number at which the fluorescence passes the fixed threshold. TaqMan CT values were converted into absolute copy numbers using a standard curve from miRNA U6. The Relative Quantitative RQ was expressed in Log10.

#### 2.2.4. Target Prediction Tools

The genes targeted by miR 320 and miR 423-5p and the metabolic pathways that are involved were identified by examining the specifically, online databases, miRDB (http://mirdb.org/miRDB/), TargetScan (www.targetscan.org), microRNA.org (https://tools4mirs.org/software/mirna_databases/micrornaorg/), PicTar (http://pictar.mdc-berlin.de), Gene cards.org and Kyoto Encyclopedia of Genes and Genomes (KEGG).

### 2.3. Statistical Analysis

Continuous variables are presented as mean and standard deviation, whereas categorical variables are indicated as numbers and percentages. Comparisons between groups were performed by the Mann–Whitney non-parametric test, in consideration of sample size and after the assessment of the normality of the data by the Kolmogorov–Smirnov test.

A *p*-value < 0.05 was considered significant. Statistical analysis was performed using Stata (18.0).

## 3. Results

Thirty-nine subjects were enrolled in this study; 20 patients were assigned to group 1 (contact lens wearers), and 19 patients were enrolled in group 2 (control group).

The demographic characteristics of these patients are summarized in Table 1. The expression levels of miR-320 and miR-423-5p were evaluated in both groups and the descriptive statistics (mean, standard deviation, and confidence interval) are presented in Table 2.

**Figure 1:**
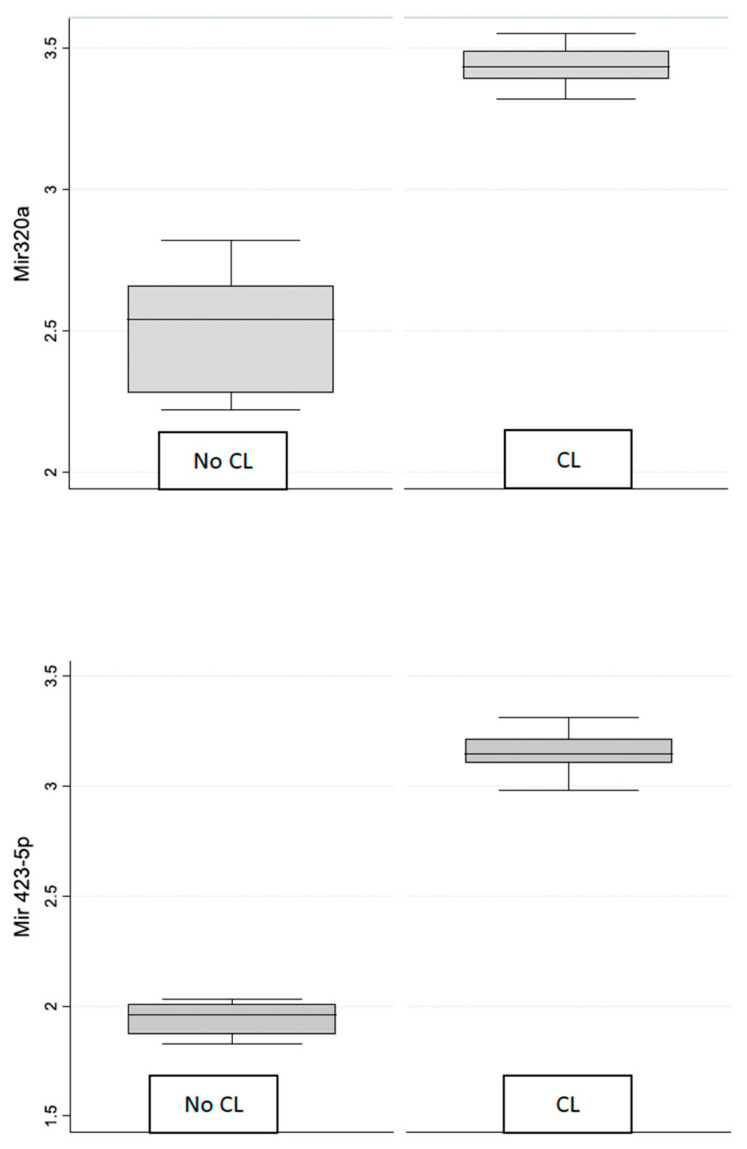
Box plot of mirR-320 and miR-4235p in control and contact lens groups.

To illustrate the differences in expression levels between contact lens wearers (CL) and the control group (No CL), the box plots were created for miR 320 and miR 423-5p (Figure 1).

The box plots clearly demonstrate that the median expression of both miR-320 and miR423-5p is higher in the CL group compared to the control group. The interquartile ranges (IQRs) indicate a wider distribution of expression values in the control group for both microRNAs, suggesting greater variability.

There were no statistically significant differences between the two groups in terms of age, sex, or refraction.

### Expression of miR-320 and miR 423-5p

The sensitivity of the method used allowed us to evaluate the gene expression of 2 microRNAs, miR-320 and miR 423-5p, in the group of selected patients. Indeed, it results in a symmetry of expression between the 2 miRNAs studied. Both miR-320 and miR 423-5p expression was significantly higher in group 1 compared to group 2 (*p* < 0.001). Specifically, the miR-320 levels showed a statistically significant difference (*p* < 0.001) between the two groups, with group 1 having a significantly higher median level than group 2. Similarly, significant differences were observed for miR 423-5p, with group 1 displaying a higher median value compared to group 2 (*p* < 0.0001).

## 4. Discussion

Recent interest in epigenetics and its possible application in ophthalmology focused on the possibility to determine the new biomarkers of ocular disorders. In recent years, microRNAs have attracted a lot of attention in the fields of tumors [4,5], retinal pathologies [6], ocular surface inflammatory processes and overall dry eye diseases [7,8], and Liao et al. highlighted the importance of the potential role of miRNAs in the therapy of dry eye diseases [8].

Ocular surface disorders represented by DED are the most frequent ocular surface pathology in developed countries with a prevalence rate ranging from 5 to 50% with several impacts over the quality of life of patients [23,24,25]. This multifactorial pathology frequently arises from environmental risk factors including CL wearing, which induces cell apoptosis and initiates the vicious circle of the dry eye disease [26,27]. Two epidemiologic studies conducted in Japan highlighted the high risk of developing dry eye symptoms in contact lens wearers [27,28,29]. Moreover, TFOS Dry Eye Workshop in 2017 recognized CL use as a risk factor for dry eye disease [24].

Different studies reported morphological ocular surface changes in extensive CL wearers, demonstrating chronic stress and significant decrease in conjunctival goblet cells as well as their impact on meibomian gland morphology [23,30,31]. Additionally, in vivo corneal confocal microscopy examination revealed higher dendritic cell density in the central cornea, in response to a chronic inflammatory stimulus induced by extensive CL use [32].

A recent study on the samples of pterygium collected from 253 patients revealed that miRNA-145 is downregulated in this ocular surface disease, and it is inversely correlated with severity, extension, and the vascular spread of the lesion [33]. Furthermore, reports on the mutations of miRNA-184 showed its involvement in the reduction of corneal thickness, leading to keratoconus, and in the development of iris atrophy and the early formation of cataracts [34,35].

To our knowledge, there are no ex vivo comparative studies on the epigenetics of corneal epithelium. In this pilot study, we investigated the expression of miR-320 and miR-423-5p in the healthy corneal epithelium of CL wearers and no wearers. The choice of these two miRNAs was related to their involvement in the processes of cellular apoptosis and chronic inflammation that occur on the ocular surface of the CL wearers and in patients with ocular surface disorders [20,36]. We were able to demonstrate the significantly increased levels of miR-320 and miR-423-5p in the corneal epithelium of CL wearers as compared to normal.

MiR-320 regulates the target gene TP53 Regulated Inhibitor of Apoptosis 1 (TRIAP1) [20]. It is involved in the modulation of the mitochondrial apoptotic pathway by ensuring the accumulation of cardiolipin in mitochondrial membranes and interacting with several proteins and complexes [36]. Particularly the TRIAP1:PRELID1 complex prevents apoptosis by the mediation of the transfer of phosphatidic acid (PA) between liposomes and probably functions as a PA transporter across the mitochondrion intermembrane space to provide PA for cardiolipin synthesis in the inner membrane [37].

MiR 423-5p o 320 regulate the target gene NFAT-Activating Protein with ITAM Motif 1 (NFAM1) [38]. MiR 423-5p is localized in chromosome 17 (17q11.2) and is involved in the modulation of genes, like NFAM1, involved in the metabolic process of inflammation [38,39]. Particularly the protein encoded by NFAM1 is a type I membrane receptor that activates cytokine gene promoters such as the IL-13 and TNF-α [40]. The encoded protein contains an immune-receptor-tyrosine-based activation motif (ITAM) and is thought to regulate the signaling and development of B-cells. Furthermore, it may function in the immune system as a receptor which activates, via the calcineurin/NFAT-signaling pathway, downstream cytokine gene promoters.

Phospholipase C β 1 (Phosphoinositide-Specific) (PLCB1) is the target gene regulated by miR 423-5p [41]. The protein encoded by this gene catalyzes the formation of inositol 1,4,5-trisphosphate and diacylglycerol from phosphatidylinositol 4,5-bisphosphate. This reaction uses calcium as a cofactor and plays an important role in the intracellular transduction of many extracellular signals.

These findings focus on the new diagnostic tool related to miRNA biomarkers with a possible targeting of the therapy in ocular surface diseases. The increased expression of miRNAs involved in apoptosis and inflammation is suggestive of the regulation of pathways controlled by the activated genes involved in the etiopathogenesis of the ocular surface disorders. To our knowledge, this is the first study of miRNA expression on ex vivo, freshly harvested human corneal epithelium. The significant CL-induced changes in miRNA expression might be considered in further investigation of the role of the environmental risk factors in the epigenetic changes in ocular surface. In this study, we have highlighted the alteration of the expression of two miRNAs involved in apoptosis and we propose to continue this research with transfection and inhibition experiments in order to understand and determinate the possible use of these MirNAs in the field of ocular surface diseases.

Additionally, further investigations of different miRNAs and using a higher number of specimens collected from patients with a history of environmental exposure and dry eye symptoms are suitable for the better determination of miRNA biomarkers for ocular surface diseases.

## 5. Conclusions

MicroRNAs have appeared on the scientific scene over the past 10 years; by playing a role in tissue homeostasis, they seem to be excellent predictive biomarkers in both normal and pathological conditions. In the present study, we report the expression of two miRNAs, known to be associated with tissue damage, in the corneal epithelial cells in the chronic ocular surface stress caused by extensive CL wear. Despite the limited number of patients analyzed, the preliminary results are a prelude to indicating the importance of mir-320 and mir423-5p in ocular surface physiology. In the near future, further studies could allow for a better comprehension of the role of these epigenetic effectors in ocular surface disorders.

## Figures and Tables

**Table 1:** Demographic characteristics of enrolled patients.

	CL	Control
N	20	19
Age (years)	30.4 ± 8.83	37.89 ± 8.87
Male/Female n (%)	5 (25%)/15 (75%)	10 (52.63%)/9 (47.37%)

**Table 2:** Mean and standard deviation of miR-320a and miR-4235p with 95% confidence intervals in CL group and control group.

Group	Variable	Mean	Std. Dev.	Conf. Interval 95%	
CL group	miR-320a	3.44	0.07	3.41	3.47
	miR-4235p	3.15	0.09	3.11	3.19
Control group	miR-320a	2.50	0.20	2.41	2.60
	miR-4235p	1.95	0.07	1.92	1.98

## Data Availability

The original contributions presented in the study are included in the article, further inquiries can be directed to the corresponding author.
